# Supplementary material for: High resolution Hall measurements across the VO2 metal-insulator transition reveal impact of spatial phase separation
Source: Sci Rep. 2016 Jan 19;6:19496. doi: 10.1038/srep19496 (PMC4726073; doi:10.1038/srep19496)
Supplement: Supplementary Information [file srep19496-s1.doc]

**High resolution Hall measurements across the VO_2_ metal-insulator transition reveal impact of spatial phase separation**

Tony Yamin^1,2^, Yakov M. Strelniker^1^ and Amos Sharoni^1,2^.

Department of Physics, Bar Ilan University, Ramat-Gan, Israel IL-5290002 Bar-Ilan Institute of Nanotechnology & Advanced Materials, Ramat-Gan, Israel IL-5290002

**Supplementary Figure 1 | Properties of VO_2_ thin film.** (a) and (b) are XRD measurement of the VO_2_ thin film: **(a)**
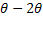
 measurement with peaks of VO_2_ and sapphire marked. There is a single peak at 37.12° corresponding to the VO_2_ (200) peak, as expected for growth on R-cut sapphire. **(b)** In-plane rotation measurement with
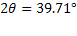
 corresponding to the VO_2_ (002) plane. Only two peaks spaced by 180° are present, corroborating epitaxial growth. **(c)** SEM image showing grain side of ~100nm. **(d)** AFM image of the VO_2_ disc edge with scale bars in the image. Inset- histogram of VO_2_ height with average height of 65nm and RMS roughness of 5nm.


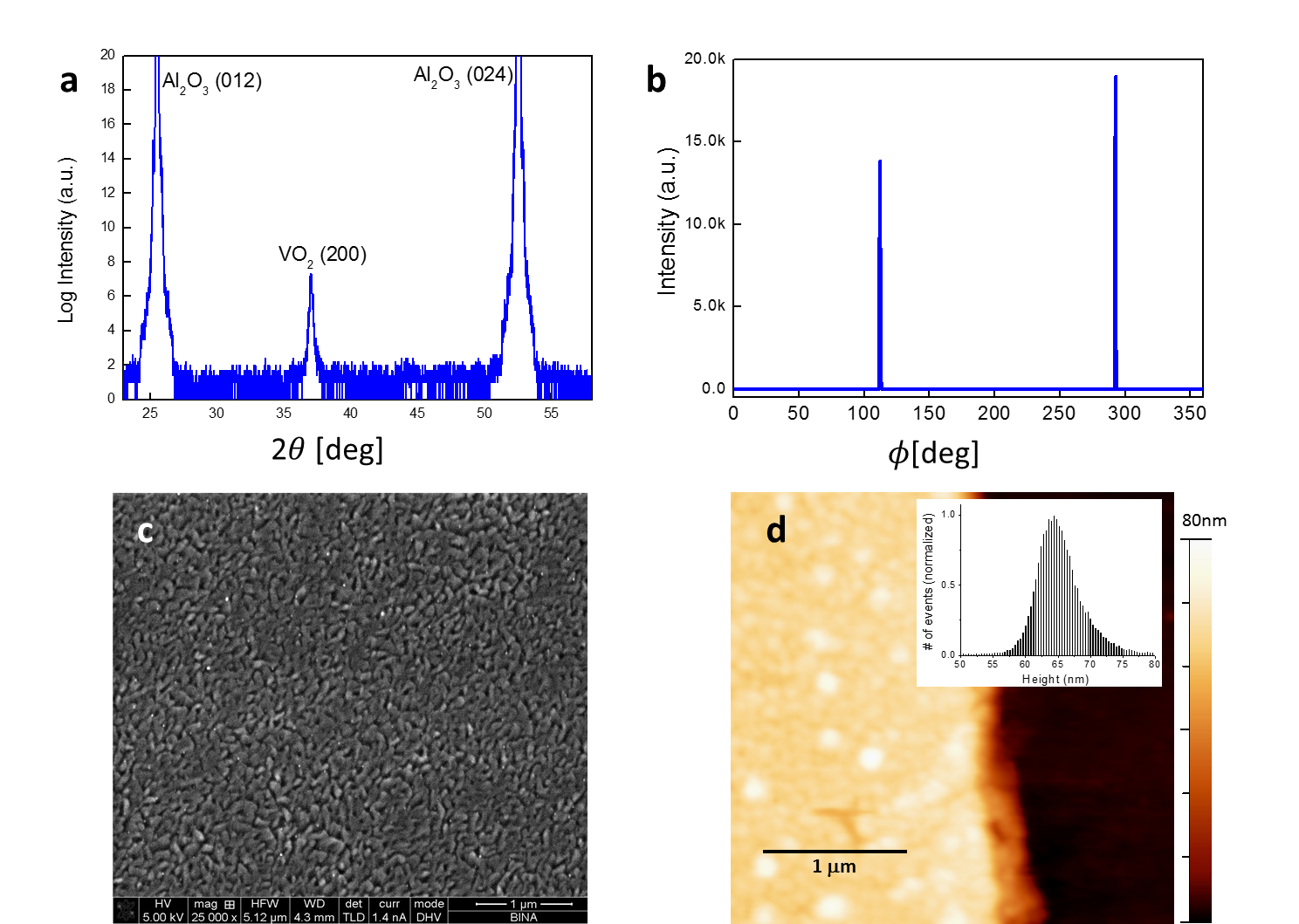


**Supplementary Figure 2 | R_T_ vs H measurement** **of the two geometries**. Transverse resistance vs. magnetic field are shown for the clover leaf geometry (red curve) and disc geometry (black curve) using the Onsager method for two temperatures- **(a)** 300K and **(b)** 340K. The Cover geometry is nosier than the disc. Possible reasons are: (i) associated with the size and geometry of the device, since in the clover geometry the resistance is larger (not resistivity). (ii) there is some damage caused by the RIE to the sample’s circumference that results in nosier measurements. We notice a difference in the Hall resistance measured at low temperatures (a factor < 2, which is very small relative to the 4 orders change during the transition). Theoretically the different geometries are not supposed to modify the results, but this may not be experimentally accurate.


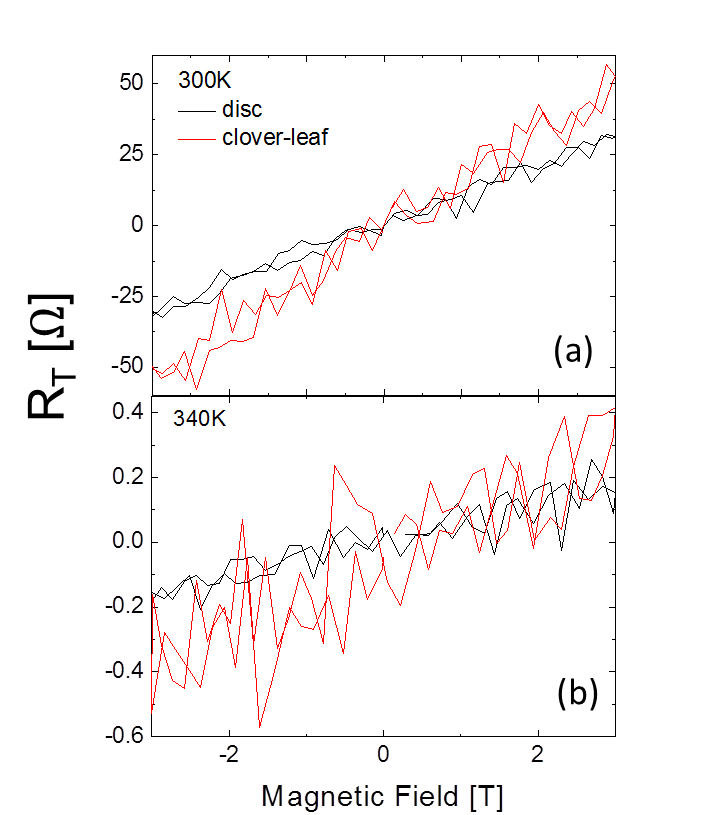


**a**

**b**

**Supplementary methods: magneto-transport measurement**

We use contacts’ numbers defined in supplementary Fig. 3g to describe measurement scheme. Hall Effect was measured in three ways. (1) A conventional Hall measurement. After stabilizing the temperature (~30 minutes) R_T_, i.e. the transverse voltage, was attained by measuring the voltage V_13_ divided by the injected current I_24_, as a function of magnetic fields between -3T and 3T. (2) Using the Onsager reciprocity relation:
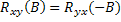
 [1-2]. We measure R_T_ by two different configurations, quickly switching between the two: first, current was injected between contacts 1 and 3 and voltage was measured between 2 and 4, then current was injected between contacts 2 and 4 and voltage was measured between 1 and 3. Since the offset from the longitudinal resistance is symmetric in field, subtracting the two measurements and dividing by 2 results in the pure transverse resistance. Also here, magnetic field was modified between -3T and 3T. (3) Using the reciprocity relation and a constant magnetic field of 8T, 0T or -8T, while sweeping the temperature.

In supplementary Fig. 3 we present results for the disk geometry showing the advantages of the reciprocity method. Supplementary Figs. 3a, 3b and 3c are of R_T_ vs. H for method (1) explained above, and at 300K, 340K and 350K, respectively. Ideally, at zero magnetic field a zero Hall-voltage is expected. But as commonly observed, R_T_ has a considerable offset. This is an outcome of even small misalignment of the transverse contact electrodes, which results in a contribution from the longitudinal resistance, R_xx_. This contribution, albeit small, could be much larger than R_T_, especially if sample resistance is high, like in the case presented here. This is further evidenced in the much smaller resistance offset for the metallic state measurement at 350K. In addition, since R_xx_ is temperature dependent especially during the MIT, measurements require that sample temperature is very stable. The difficulty is exemplified in supplementary Fig. 3b) where even after a 20 minute stabilization period the change in longitudinal resistance makes Hall measurements impossible. Measurements for the reciprocity method (2) of the same three temperatures are shown in supplementary Figs. 3(d)-3(f). These measurements are much less noisy, the offset is virtually zero while the slopes do not change. Now, there is a clear linear slope at T=340K (supplementary Fig. 3(e)) which is during the MIT. Additionally, the sudden jump in supplementary Fig. 3(c) disappears in supplementary Fig. 3(f), indicating it originates from the longitudinal resistance.

**Supplementary Figure 3 | R_T_ vs. H for 3 temperatures of 300K, 340K and 350K.**  **(a-c)** Standard measurement **(d-f)** measurement using the Onsager method. Temperature and method are marked in image. **(g)** Device configuration and electrode numbering used to define measurement scheme.


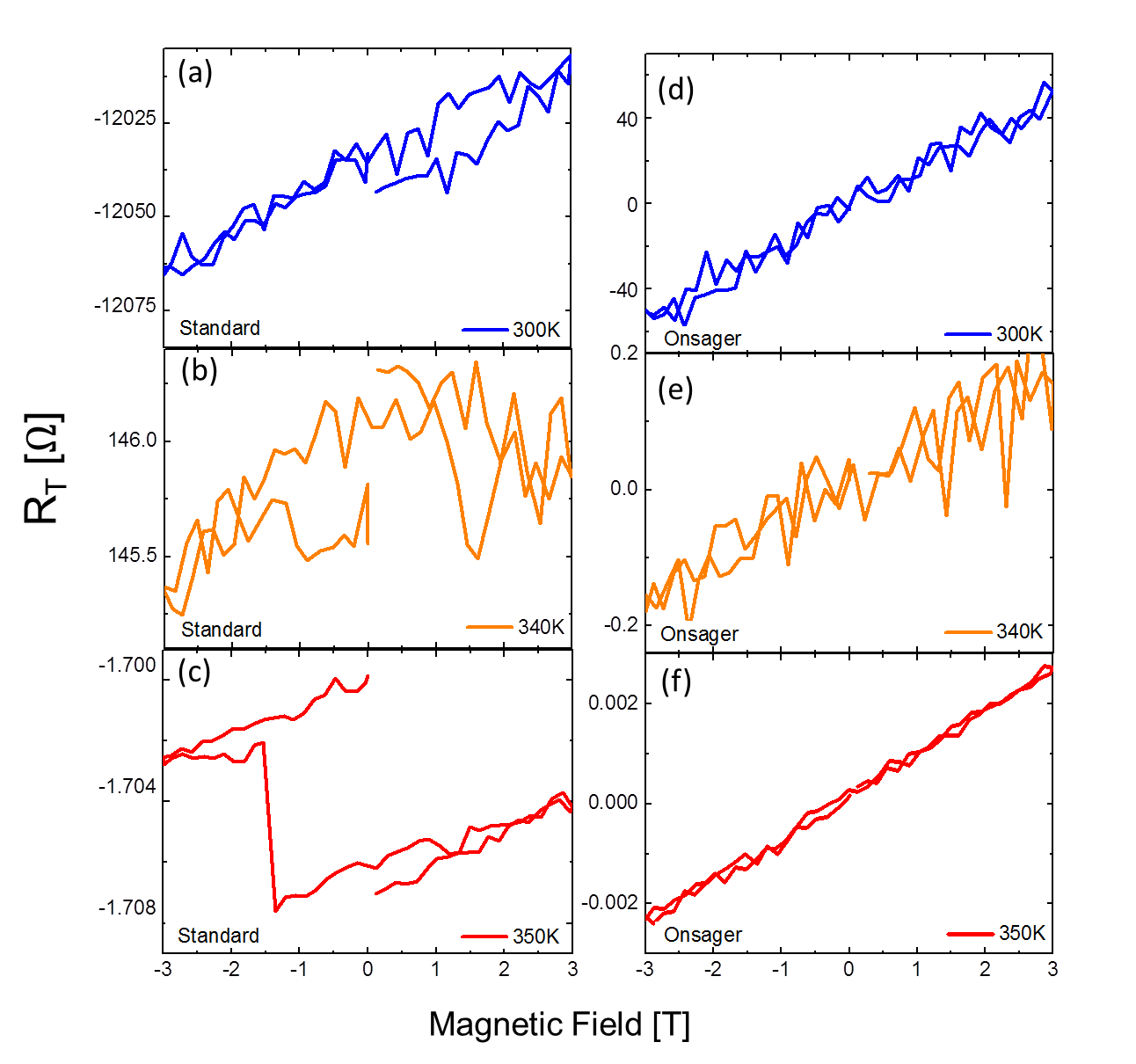


**g**


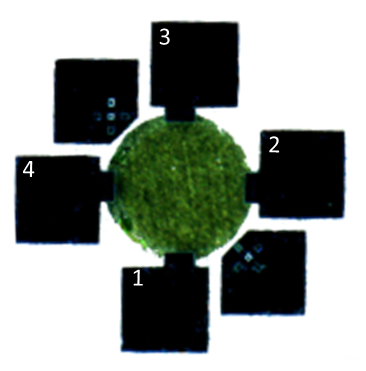


**Supplementary Figure 4 | Measurements of the resistance and the Hall resistance vs. temperature. (a)** Continuous longitudinal resistance measurements which overlap for all three fields of 8T (blue curve,) -8T (black curve) and 0T (red curve), depicting the reproducibility of the sequence and lack of magnetoresistance in the sample. **(b)** Hall resistance at different constant magnetic fields of 8T (blue curve,) -8T (black curve) and 0T (red curve).The small difference from zero for the 0T measurement comes from the subtraction of consecutive measurements, and since it is relatively small it does not affect the results.


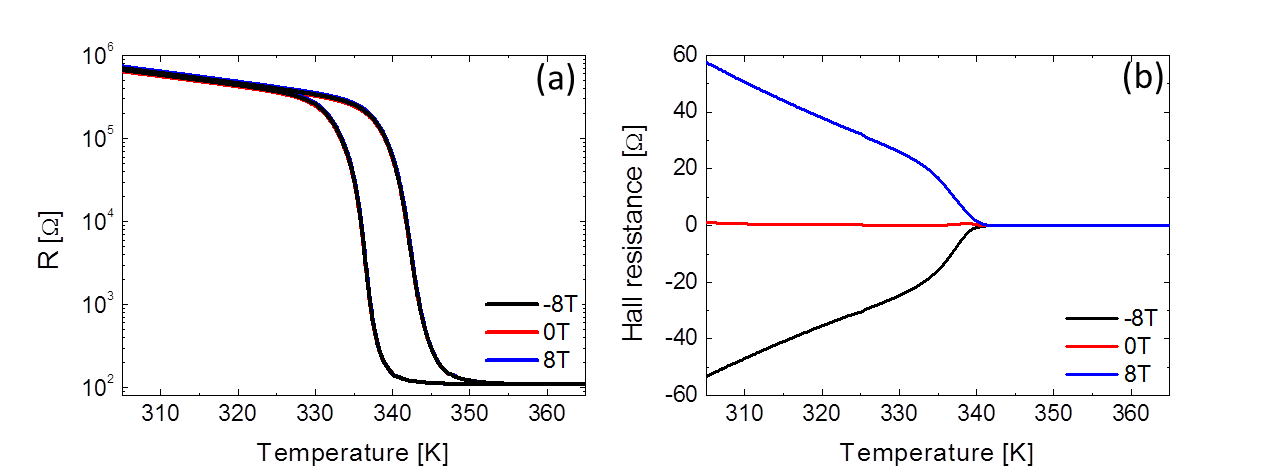


**Supplementary Figure 5 | The ‘naively’ extracted charge carrier density** **vs. temperature.** Carrier density naively extracted from
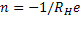
 assuming one carrier type and that Hall mobility is actual mobility, enabling comparison with previous reports.


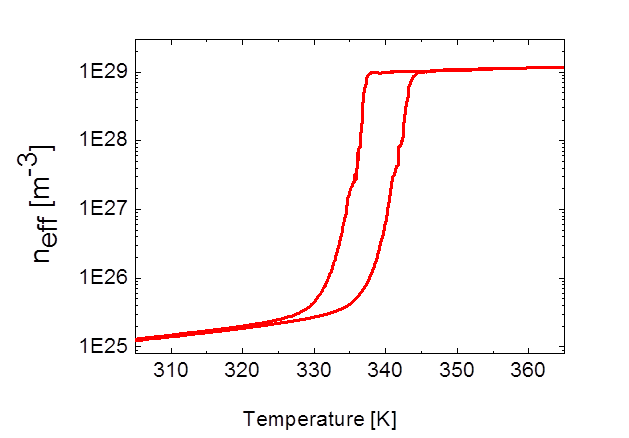

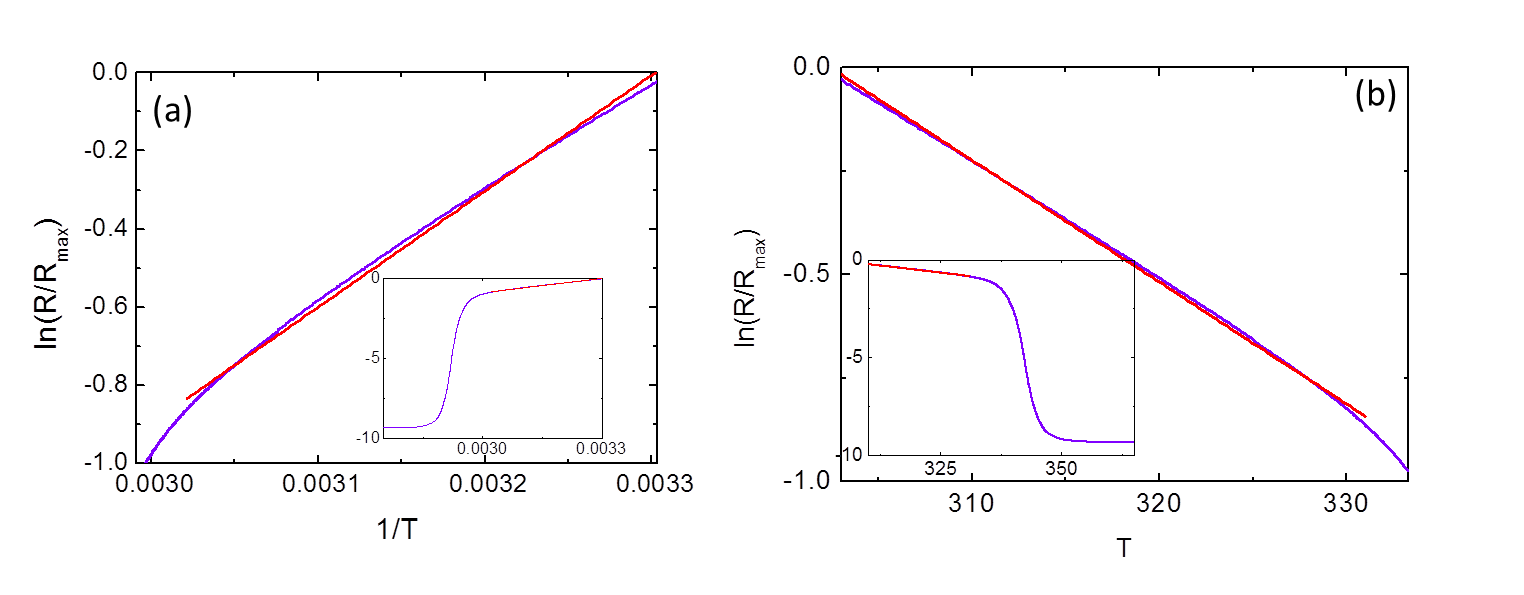


**Supplementary Figure 6 | Extrapolating semiconductor and metal phase properties for exact relation analysis.** When analyzing the results during the MIT we used properties extrapolated from the temperature regime where there is a single phase. For the metallic phase we use a constant resistance measured above 355K equal to 100
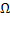
. Similarly, we extracted the Hall coefficient from the corresponding Hall measurement resulting in a metallic carrier density of
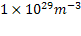
. The semiconductor phase behavior was extrapolated from its single phase properties at temperatures between 300 and 327K. In general, we expect the semiconductor resistance to follow:
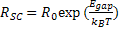
. In **(a)** we plot ln(R/R_max_) vs. 1/T and corresponding linear fit (inset shows full range). **(b)** We attempted fitting the data also to the function:
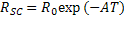
, where A is a fitting constant. Interestingly, this function seems to agree better with the results. We compared the results of the exact relation theorem using both functions and found them to result in almost identical final results. We extrapolated the Hall coefficient using the same function. This actually means that we are assuming the SC mobility is constant across the transition and does not depend on the fraction of SC domains, i.e. the SC carrier density and mobility even for small domains correspond to the bulk properties.

Supplementary Reference

[1] L. Onsager, Phys. Rev. **38**, 2265 (1931).

[2] H. Casimir, Rev. Mod. Phys. **17**, 343 (1945).
